# Supplementary material for: Complexome profiling of the Chlamydomonas psb28 mutant reveals TEF5 as an early PSII assembly factor
Source: Plant Cell. 2025 Mar 17;37(6):koaf055. doi: 10.1093/plcell/koaf055 (PMC12164590; doi:10.1093/plcell/koaf055)
Supplement: koaf055_Supplementary_Data [file koaf055_supplementary_data.zip › psb28_interactive.zip!/plots/intensity/qualityPlots/peptides/HSP90B_Cre02.g080650.t1.2.html]

### Cre02.g080650.t1.2

Trivial name: HSP90B  
SeqCoverage [median]: 21.90  
Mass: 93.12 kDa  
  
 

| Sequence | MissedCleavage | Mass [Da] | wtIntensity | muIntensity | Score |
| --- | --- | --- | --- | --- | --- |
| AQAFSRPGSSFTPTQR | 1 | 1736.86 | 476.28 | 3351.74 | 71.98 |
| GIVDSDTLPLNVSR | 0 | 1484.78 | 896.70 | 3714.79 | 103.21 |
| KPSDVTEEEYQK | 1 | 1451.68 | 321.95 | 596.31 | 132.79 |
| LAAATEETVEESAVATAR | 0 | 1817.90 | 0.00 | 687.95 | 67.69 |
| LGIIEDTTNR | 0 | 1130.59 | 666.83 | 1116.16 | 76.83 |
| NLGTIAK | 0 | 715.42 | 0.00 | 706.03 | 70.00 |
| SGTSAFLEQMQK | 0 | 1325.63 | 0.00 | 535.18 | 96.33 |
| SILGDGDTSNLEIK | 0 | 1460.74 | 485.61 | 0.00 | 58.68 |
| TGDQLTTLDEYIGR | 0 | 1580.77 | 314.55 | 2566.26 | 137.88 |
| TLEINPR | 0 | 841.47 | 1756.69 | 3811.89 | 105.39 |
| VDNGVSSGHATATDATSIHR | 0 | 1994.94 | 0.00 | 714.22 | 38.45 |
| VVDDSKLQGVK | 1 | 1186.66 | 355.99 | 0.00 | 48.44 |
|  |  |  | 5274.6 | 17800.5 |  |
